# A narrative on diversity, equity, and inclusion in surgery: insights from the European Society of Coloproctology and identification of points for action

**DOI:** 10.1007/s13304-023-01685-3

**Published:** 2024-01-27

**Authors:** Zoe Garoufalia, Vittoria Bellato, Miguel F Cunha, Nicolas Avellaneda, Nagendra N Dudi-Venkata, Cristián Gallardo, Jeremy Meyer, Gloria Zaffaroni, Peter Christensen, Erman Aytac, Richard R W Brady, Gianluca Pellino, the European Society of Coloproctology ESCP Operation Equal Access Working Group

**Affiliations:** 1 Ellen Leifer Shulman and Steven Shulman Digestive Disease Center, Cleveland Clinic Florida, Weston, FL USA; 2 ESCP Social Media Committee Co-Chair, ESCP, Portsmouth, United Kingdom; 3 Minimally Invasive Surgery Unit, Università di Tor Vergata, Rome, Italy; 4 Gastroenterology Surgery Department, San Raffaele Hospital, Milan, Italy; 5 Surgical Department, Algarve University Hospital, Portimão, Portugal; 6 Nueva Proctologia, Buenos Aires, Argentina; 7 CEMIC, Buenos Aires, Argentina; 8 Danish Cancer Society National Research Centre for Survivorship and Late Side Effect to Cancer in the Pelvic Organs, Department of Surgery, Aarhus University Hospital, Aarhus, Denmark; 9 Colorectal Unit, Department of Surgery, Royal Adelaide Hospital, Port Road, Adelaide, 5000 Australia; 10 Adelaide Medical School, Faculty of Health and Medical Sciences, University of Adelaide, Adelaide, Australia; 11 Servicio de Coloproctologia, Hospital Clínico San Borja Arriarán, Santiago, Chile; 12 Division of Digestive Surgery, University Hospitals of Geneva, Rue Gabrielle-Perret-Gentil 4, 1211 Genève 14, Switzerland; 13 Medical School, University of Geneva, Rue Michel-Servet 1, 1205 Genève, Switzerland; 14 Department of General Surgery, Hospital L. Sacco, Milan, Italy; 15 Department of Surgery, School of Medicine, Atakent Hospital, Acibadem Mehmet Ali Aydinlar University, Instanbul, Turkey; 16 Incoming ESCP Communication Committee Chair, ESCP, Portsmouth, United Kingdom; 17 Newcastle Centre for Bowel Disease Research Group, Newcastle Upon Tyne Hospitals NHS Foundation Trust and Newcastle University, Queen Victoria Road, Newcastle Upon Tyne, United Kingdom; 18 ESCP Communication Committee Chair, ESCP, Portsmouth, United Kingdom; 19 Colorectal Surgery, Vall d’Hebron University Hospital, Universitat Autonoma de Barcelona UAB, Barcelona, Spain; 20 Department of Advanced Medical and Surgical Sciences, Universitá Degli Studi Della Campania “Luigi Vanvitelli, Naples, Italy

**Keywords:** Inclusion, Diversification, Diversity, Equity, Interview, Opinion leaders, Role models

## Abstract

The focus of the 2022 European Society of Coloproctology (ESCP) annual campaign was diversity, equity, and inclusion (DEI) in surgery. The ESCP “Operation Equal Access” campaign sought to interview key-opinion leaders and trainees, to raise awareness on inequalities, inform the community of the status of the topic, and to identify future areas for improvement. The ESCP Social Media Working Group interviewed experts who have made significant contributions to DEI in colorectal surgery and were acknowledged opinion leaders in the field. The interviews focused on their career, professional life, experiences, and opportunities during their training, and their views on DEI in colorectal surgery. DEI principles, education, and values need further promotion to reduce and address bias within the profession and overall improve the experience of minority community including health professionals and patients. International Societies are working to facilitate training opportunities and overcome DEI, and networking have contributed to that. Collaborations between societies will be pivotal to contribute to offering research and leadership opportunities equally. Access to advanced workshops including cadaveric training and simulation can be consistently promoted and provided globally via societies through telemonitoring. Involving patients in research should be encouraged, as it brings the perspective of a living experience.

## Introduction

In June 2022, the #OperationEqualAccess campaign was launched by The European Society of Coloproctology (ESCP). The campaign focused on diversity, equity, and inclusion (DEI) in surgery to address how disparities in the medical sector affect career progression, well-being, and, more importantly, patient care, as well as to promote initiatives helping to remove these barriers.

Although it may be reasonably expected for the healthcare profession to incorporate such values in the workplace, disparities remain prevalent, especially in medical academic and leadership positions [1–3]. The differing professional opportunities for surgeons relating to their gender, race, socioeconomic status, and sexual orientation were previously reported by both trauma [4] and colorectal surgeons [5]. Most importantly, these disparities are increasingly recognized and some societies are issuing action plans on DEI, such as the American College of Surgeons [6], the American Society of Colon and Rectal Surgeons [7], and the Association of Coloproctology of Great Britain and Ireland [8].

To explore the current situation, the ESCP conducted several interviews with key-opinion leaders (KOLs). In this report, we summarize those interviews and draw conclusions about the current status of DEI in general and colorectal surgery, aiming to identify those future initiatives and actions that could reduce disparities and promote DEI.

## Methods

In June 2022, the Social Media Subcommittee of the ESCP decided to make DEI the theme of its annual campaign. The campaign was titled *#OperationEqualAcess*, during which, the ESCP Social Media Working Group interviewed KOLs on their perspectives on DEI in general and colorectal surgery, and how the society can move forward toward achieving that in our specialty.

### Description of the project and aims

This article is a qualitative narrative analysis of the interviews carried out within the scope of the ESCP #OperationEqualAccess campaign. Interviewees were asked to provide evidence and their view on three specific themes: diversity, equity, and inclusion within the general and colorectal surgery field. This report is intended to be a narrative description of the information and content shared by those experts during the interviews, and the eventual aims were (1) to define the outstanding issues in DEI, (2) to fill research gaps as sound evidence is hardly available, and (3) to suggest strategies to tackle the issues [9].

### Workflow and selection of the interviewees

The ESCP Social Media Working Group, which in itself is a diverse group of colorectal surgery trainees and consultant surgeons, selected KOLs in the field. The criteria used to identify the KOLs included (1) expertise in general or colorectal surgery or being a stakeholder in the field, (2) interest in research or proven academic career, and (3) previous work on DEI. The interviewees were selected and agreed on by the ESCP Social Media Group in collaboration with the ESCP Executive Committee, either based on their publicly known work in terms of DEI in the field of colorectal surgery or based on their personal example, as some of them blazed the way, overcoming adversities in this field and leading the way for next generations. Attention was paid to ensure adequate representation and diversity of the KOLs, to address the perspectives and point of views of all stakeholders. Factor related to geographical, seniority, gender, and profile aspects were taken into account.

The interviews were structured taking into account the role of each Interviewee among the scientific community, their background as regards promoting diversity (e.g., previous publications or activities, representatives of DEI at societies, trainees involved in equal access). Each ESCP Social Media Working Group member was assigned an Interviewee and was in charge of conducting the interviews and providing the team with the summary. All interviews were structured to include questions focused on (1) interviewees’ career and professional life, (2) experiences and opportunities during their training, and (3) views and work on DEI in colorectal surgery.

All interviewees agreed to be mentioned and allowed the use of the content of their interviews for the development of this manuscript.

**Fig. 1:**
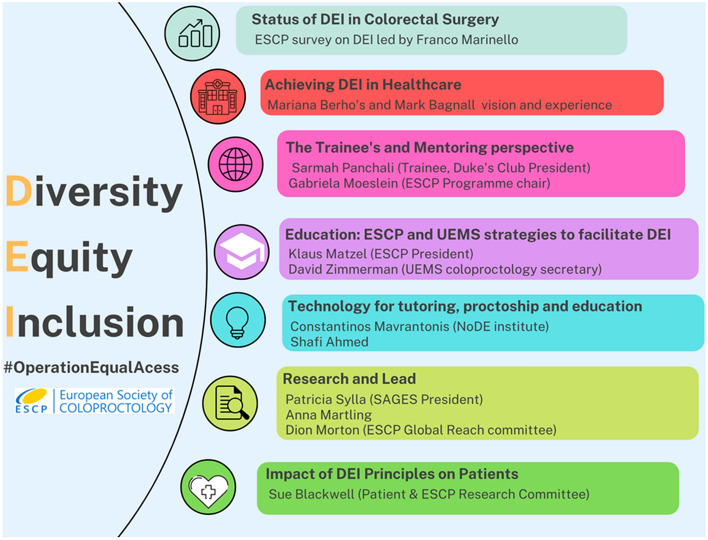
Topics covered during #OperationEqualAccess

The interviews are presented and grouped in the manuscript based on the topic covered (Fig. 1), and the full scripts and recordings can be accessed through the links provided in the Supplementary Material.

### Definitions

*Diversity*: All possible ways and characteristics that can distinguish any person from another are included in the broad term of diversity. Thus, diversity is a fact; it does not require action but awareness. People can differ in many ways, such as race, socioeconomic status, gender, age, mental and physical abilities, ideas, perspectives, or values.

*Equity*: Equity, on the other hand, is often confused with equality. Equity, an Aristotelian concept, guarantees fair treatment, access, and equality of opportunities by removing barriers that would have precluded access for some individuals from certain groups.

*Inclusion*: Inclusion is an act ensuring that this diverse, wide range of individuals feel welcomed and part of a team. This includes any practice or policy aimed to offer opportunities for those who might otherwise be marginalized.

## Results

### Status of DEI in colorectal surgery

In a survey, supported by the ESCP, Marinello et al. found that more than half of female surgeons surveyed reported gender discrimination, with almost half (44%) of the participants not feeling equally respected in the workplace because of their race, gender, or sexual orientation [5]. DEI in the health workforce is crucial to gaining fresh perspectives, but many colorectal surgeons face barriers daily [5, 10]. [https://www.escp.eu.com/news/2399-operationequalaccess-dr-franco-marinello-interview].

### Achieving DEI in healthcare

Achieving a diverse medical staff is not sufficient, it is also necessary to encourage inclusion. People with different identities need to feel valued within a team or workplace. A conscious effort is imperative to achieve staff diversity, and this effort starts from the job description when the position is advertised. Certain aspects of a job description that might stimulate the application from minority groups may include words such as flexibility of schedule, full- or part-time employment options, and LGTBQ + members should consider applying. [https://www.escp.eu.com/news/2418-operationequalaccess-dr-mariana-berho].

Mistreatment is common among LGBTQ + trainees, who have twice as high likeliness of considering quitting their job. [11] LGBTQ + surgeons in training should be supported and encouraged by senior surgeons who are part of the LGBTQ + community. Progress had been made in the United Kingdom, and organizations like ESCP can potentially eliminate bias and promote the adoption of fairness by building bridges across European nations and thus enabling change. Any progress takes persistence and effort, and ESCP can be the driving force and the voice of change. The same can apply to other International Societies as well [https://www.escp.eu.com/news/2428-operationequalaccess-mark-bagnall-interview]. It is desirable that societies have a strategy on DEI as part of their mission, and set measurable outcomes to verify any progress made and areas for improvement. As a matter of fact, the ESCP have recently established a “Diversity, Equality and Inclusivity Working Group” that will also work on launching and improving the implementation of DEI initiatives and measuring their impact.

### The trainees' perspective and mentoring to overcome barriers

Diversity provides new perspectives on achieving aims, new approaches to tackling a problem, and, as a trainee, new methods to learn. There might be those that are not aware of their actions’ consequences because they have not experienced bias themselves, been educated in DEI principles or have not been part of a diverse environment. Further steps and measures are in progress at an organizational level, like governments, associations, institutions, and societies, with Dukes' Club being a forward-thinking example. In fact, the Duke’s Club of the Association of Coloproctology of Great Britain and Ireland (ACPGBI) achieved a highly visible DEI policy and instituted closed voting and anonymized applications for prizes, courses, and committee positions. [https://www.escp.eu.com/news/2411-operationequalaccess-ms-panchali-sarmah-interview].

Role models are an essential part of the change, and it is crucial for mentors to support younger fellows actively. Multi-generational mentorship could assist in tackling many difficulties as mentoring is a two-way system where each component can learn from the other [https://www.escp.eu.com/news/2419-operationequalaccess-prof-gabriela-moeslein-interview]. Mentorship plays a critical role in surgical training as well as the feeling of inclusion, positive work environment, and the possibility of progress. Surgeons must address the potential for gender bias, and work culture or commitments should not determine one’s decision to have a family. It has been recently found that surgeons are offered inadequate parental leave, with relevant disparities across different countries [12]. Younger surgeons who become parents should look for help and create a network to cope with this highly demanding lifestyle. [https://www.escp.eu.com/news/2417-operationequalaccess-prof-anna-martling-interview].

### Education: ESCP and the Union Européenne des Médecins Spécialistes (UEMS) are working to facilitate DEI

Societies can improve equity in educational opportunities, and the policy of the ESCP in this context offers valuable insights. The ESCP contributed to DEI via a permanent and accessible online teaching platform that ensures a source of education, after the annual conference for the colorectal community at large and not limited by ESCP membership. The promotion of global international fellowships, the investment in global collaborative studies, and the effort made by the ESCP program committee to promote inclusion and diversity are specific examples of the effort made by ESCP to break these barriers [https://www.escp.eu.com/news/2420-operationequalaccess-prof-klaus-matzel-interview] Academic institutions should contribute to the cause of DEI. In 2021, the ESCP working group [13] showed gender disparities in representation at conferences and participation in societies, with fewer women attending the annual conferences, being invited to serve as chairs or as speakers, and with a minor representation on committees. Male surgeons should recognize their prejudice and consider that they might not be as open to diversity as they claim to be (or think they are). On the other hand, female colleagues must also step up, accepting the invitation and supporting fellow female colleagues. [https://www.escp.eu.com/news/2412-operationequalaccess-prof-david-zimmerman-interview].

### Facilitating access to specialized postgraduate tutoring, proctorship, and global education through technology

An example of equal access to training opportunity is provided by the Network of Doctors’ Education (NoDE) Institute, a not-for-profit organization for specialized postgraduate tutoring and proctorship. NoDe is about a five-step training course in colorectal surgery, including spending time at the department, scrubbing-in, telementoring, surgical coaching for trainees' first cases, cadaveric transanal dissection, and robotic colorectal courses. Over 124 physicians have taught courses in NoDE in the last 4 years, and 878 surgeons have received free practical training. This initiative is based on doctors volunteering to train doctors [https://www.escp.eu.com/news/2405-operationequalaccess-dr-constantinos-mavrantonis-interview]. At a time when digital surgery is becoming an essential part of everyday practice of surgeons [14], innovation and technology could play a vital role in ensuring equal access to surgery [15]. The pandemic changed the mindset across the entire medical sector allowing for the extended use of telementoring and telemedicine. Access to technology is essential to capture and share knowledge and training with people remotely and equitably. Therefore, access to mobile networks and connectivity, in general, is an invaluable opportunity to remove barriers [https://www.escp.eu.com/news/2402-operationequalaccess-prof-shafi-ahmed-interview]. Active involvement of surgeons and entities is crucial to ensure a paradigm shift [16].

### Research and leadership

Engaging surgeons at a global level in research and educational initiatives is important to offer equal access to research opportunities, and it also ensures the delivery of high-quality studies, whose findings can easily be applied across the different countries. [17] This applies to different surgical specialties and to different conditions [18–20].

The ESCP Global Reach committee has been established with the aim to link surgical societies all over the world. The committee has successfully coordinated research across 50 countries, including different patients and conditions. Some current examples are the EAGLE [21] and Damascus studies [22]. Both these studies have had major participation from centers outside Europe, breaking the old paradigm of mainly European involvement and providing the opportunity for global inclusion not only for surgeons/trainees but also for patients and stakeholders. [https://www.escp.eu.com/news/2413-operationequalaccess-prof-dion-morton-interview].

Changing needs leaders, who should serve as a role model promoting DEI and leading by example. A significant mismatch can be felt between the diversity of the population we serve as physicians and the diversity within the faculty and trainees. This problem requires continuous attention and intentional actions regarding recruitment, retention, support, and promotion of a diverse environment of people in leadership positions. The Society of American Gastrointestinal Endoscopic Surgeons (SAGES) can provide an example of an entity that is deeply committed to addressing inequality in training and research [https://www.escp.eu.com/news/2422-operationequalaccess-dr-patricia-sylla-interview].

The lack of funds provided to junior researchers to start their careers represents a critical barrier that needs to be addressed promptly [23]. However, trainee-led, international, research initiatives have demonstrated that early-stage surgeons and medical students can deliver high-quality studies consistently [24, 25]. It is pivotal to truly value DEI and live by an example in real-life clinical practice rather than representing it symbolically [https://www.escp.eu.com/news/2400-operationequalaccess-julio-mayol-interview].

### Impact of DEI principles on patient management and outcomes

Involving patients in research is very important—or, better said, mandatory—because it brings the perspective of a living experience. Patients can perceive as frustrating when expected or relevant outcomes differ between researchers and patients. Patients should participate in the development of study protocols, including guidelines, and the use of patient-reported outcome measures (PROMs) [26] should be set as a priority and become a common practice for surgical research, to make the findings relevant to the patients. This goes beyond the concept of “patient participation”, introducing the concept of “patient integration”, which has been proven to be possible and successful [27, 28]. Furthermore, it is important to disseminating research results among patients with a lay version to fully include the patient in the project from project conception to reporting results [https://www.escp.eu.com/news/2404-operationequalaccess-professor-sue-blackwell-interview].

The interviewees’ background and main messages are summarized in Table 1.

**Table 1:** Summary of main messages delivered by persons interviewed during the campaign

Interviewee	Key messages	Background of the Interviewee and contribution
Shafi Ahmed	“Access to technology is essential to capture and share knowledge and training with people remotely and equitably”	Colorectal surgeon, educator, and innovator, highlighted the role of technology in improving access to education
Mark Bagnall	“The most effective way to overcome barriers is by being an example of change”	Consultant Colorectal Surgeon, shared views about DEI and the LGBTQ + Community
Mariana Berho	“A conscious effort is imperative to achieve staff diversity, and this effort starts from the job description when the position is posted”	Chairman of the Department of Pathology and Laboratory Services and Chief of Staff at Cleveland Clinic Florida shared her experiences in achieving DEI in the medical sector
Sue Blackwell	“Involving patients in research brings the perspective of a living experience”	Patient and Public Involvement representative and a member of the ESCP Research and Cohort Study Committee, shared the patient’s perspective
Franco Marinello	“Gender discrimination is common among colorectal surgeons, as well as lack of respect because of race, gender, or sexual orientation”	Consultant colorectal surgeon at the Vall d'Hebron University Hospital in Barcelona, leader of a survey on diversity bias in colorectal surgery
Anna Martling	“All surgeons must address the potential for gender bias.”	Professor of Surgery at Karolinska Institute and Chief physician and colorectal surgeon in Tema Cancer, Karolinska University Hospital, discussed the role of mentorship on DEI
Constantinos Mavrantonis	“Doctors volunteering can provide equal access to high quality training”	Colorectal surgeon, Director of the 6th Department of Surgery—Hygeia Hospital, Greece, and a Member of the Hospital's Scientific Board, shared his vision and effort through the Network of Doctors' Education (NoDE) Institute
Klaus Matzel	“The Society’s aim is to address the significant barriers to equality like institutional, political, and financial barriers and limited access to information. Training and education in Medicine are lifelong projects”	ESCP Past-President, discussed how ESCP had improved equity in educational opportunities
Julio Mayol	“The only and the easiest way to overcome prejudice is to be connected to others, to be able to see different cultures. We need to switch from an activity-based surgery to a values-based surgery”	Professor of Surgery, Hospital Clinico San Carlos, mentioned lack of funds provided to junior researchers to start their careers
Gabriela Moeslein	“We are in this together; this is not about women, not about gender, this is about our passion for surgery and loving our job”	ESCP Program Committee Chair, focused on the importance of having role models
Dion Morton	“Novel research models are providing the opportunity for global inclusion not only for surgeons/trainees but also patients and stakeholders”	Chair of the Global Reach committee, explained how the committee aims to link surgical societies all over the world
Panchali Sarmah	“Notice what is going on, and if you see something potentially unfair, ask yourself if you would be ok if that were happening to you or a family member” “There may be people that are not aware of their actions’ consequences because they have not experienced bias themselves or have not been part of a diverse environment”	Higher Specialty Trainee and President of the Duke’s club gave an insight on how diversity brings about new ways of seeing things
Patricia Sylla	“There is a significant mismatch between the diversity of the population we serve as physicians and the diversity within the faculty and trainees” “This issue requires continuous attention and intentional actions regarding recruitment, retention, support, and promotion of a diverse environment of people in leadership positions”	Colorectal surgeon, President-Elect of the Society of American Gastrointestinal and Endoscopic Surgeons (SAGES), Professor of surgery at Mount Sinai hospital in New York City, shared her experience as a role model promoting DEI and leading by example
David Zimmerman	“Before agreeing to participate in a panel, it is essential to ask about the diversity of the participating panelist and consider declining if diversity is lacking” “practice what you preach”	Secretary of the UEMS division of coloproctology, ESCP executive member, and Consultant Colorectal Surgeon at Elisabeth-Tweesteden Hospital in Tilburg, provided insights on gender disparities in academic surgery

*DEI* diversity, equity, and inclusion, *ESCP* European society of coloproctology, *UEMS* Union Européenne des Médecins Spécialistes

## Discussion

Several issues regarding DEI were identified in general and colorectal surgery. This specific theme is trending in all medical specialities, as demonstrated by the extensive efforts and actions produced by different societies and groups to address this matter [6–8, 29, 30]. The importance of this topic is further highlighted by increasing reports on the lack of diversity that can be found in the literature. This study provided important insights that need to be adequately and rapidly addressed.

A survey [5] found that almost half of the participants did not feel equally treated in their jobs. And it is more likely, that this is just the tip of the iceberg, as there are many other reports that demonstrate that DEI problems are an ongoing issue in our profession. Recently, Seehra et al. reported that the people contending for two important scientific prizes in the UK and Ireland (the Moynihan and Patey prizes) were mostly white males, with only 19% being females [31].

**Fig. 2:**
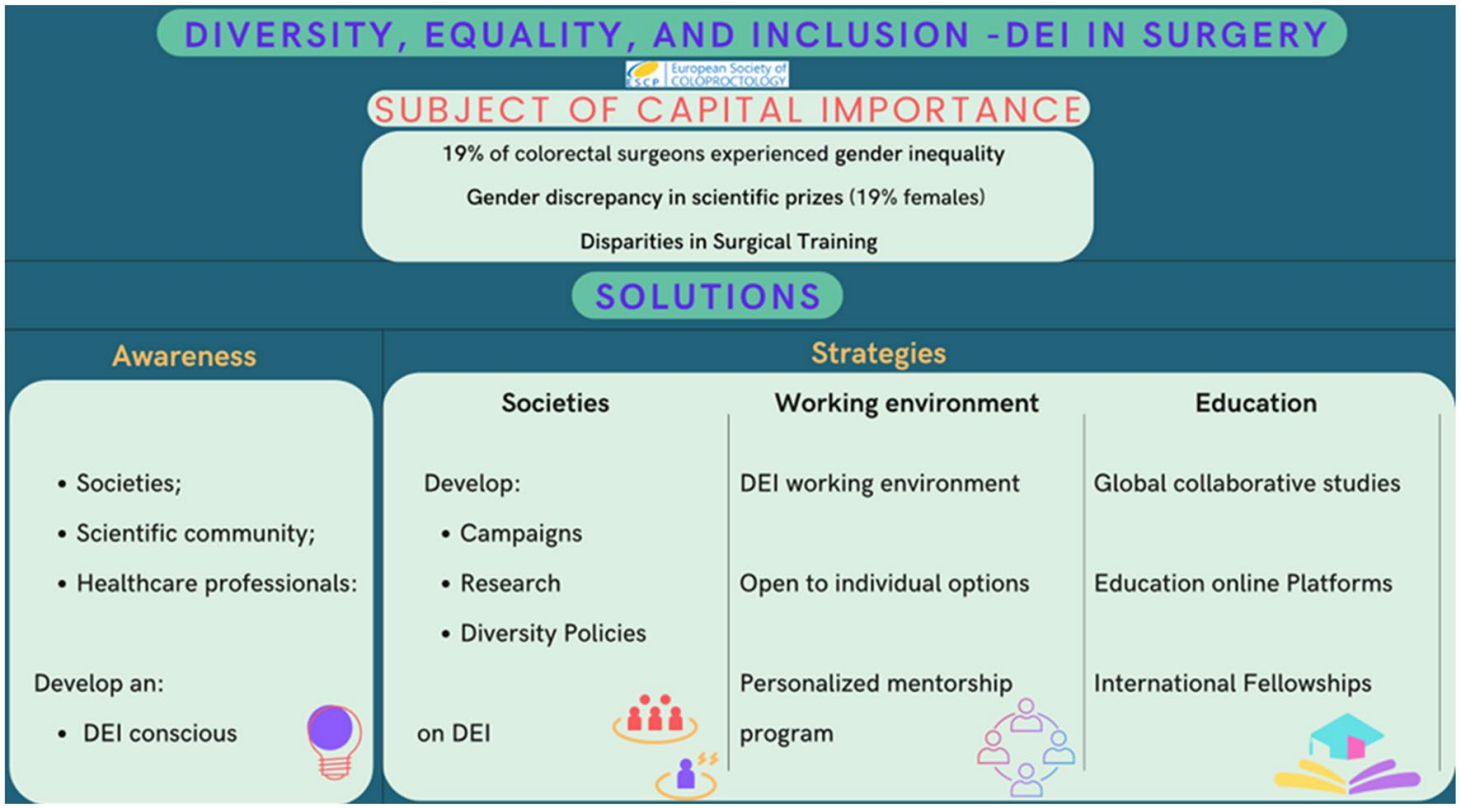
Infographic showing the strategies to promote an inclusive and diverse working environment

The campaign delivered important messages to raise awareness on strategies to tackle these inequities. One of the strategies is for organizations and institutions to endorse and practice diversity policies directly [32] (Fig. 2). This has also been suggested as a strategy by other organizations recently, including the 2020 Task Force on Diversity, Equity, and Inclusion Report of the American Society of Vascular Surgery, aiming to provide clear directions and strategies to promote an inclusive environment. Indeed, the ESCP has formed a new working group to address this topic, tasked with reporting recommendations by early 2023. The ambition is to move beyond just gender and geographical diversity.

Disparities in surgical training are an ongoing problem, with some recent reports highlighting inequality during the selection process of new trainees as well as during their learning experiences [33]. To this extent, as mentioned by the experts involved in this campaign, a good strategy would be to tackle obstacles aiming to promote a more open working environment and also to establish a personalized mentorship program that allows trainees to discuss and raise concerns through confidential channels [34] (Fig. 2).

The efforts of ESCP to extend the boundaries of its educational platforms and training opportunities beyond the European surgeons/trainees and to allow surgical community from low- and middle-income countries to also benefit from them is an evolving concept that is being reproduced in other associations [35]. One such example is the conduct of ESCP global collaborative studies involving lower income countries’ participants, which pave the way to train the surgical trainees in research methodology and reduce differences in how research studies are conducted across wider geographical locations (Fig. 2). Also, the European School of Coloproctology (ESC) and Education Committee are aiming to further reduce obstacles to educational content access. In this prospective, free educational content is offered to the ESCP audience (and not only), and delivery platforms of this content are being modified to reduce costs for the participants; the latest example is the minimally invasive Complete Mesocolic Excision “CME”’ program that is now traveling to various countries, to deliver content within each country.

## Conclusion

Disparities in DEI in general and colorectal surgery are being acknowledged and action plans are being developed by several societies to address this problem. The ESCP has stood up to make sure DEI is promoted and implemented into training and practice of colorectal surgery at a global level, which will be within the remits of their newly established DEI Working group. However, there is still a long way to go to overcome inequities in all areas of colorectal surgery, from research, training, educational opportunities, and patient care.

## Data Availability

No data were generated during the study.

## References

[1] Wehner MR, Nead KT, Linos K (2015). Plenty of moustaches but not enough women: a cross-sectional study of medical leaders. BMJ.

[2] Fang D, Moy E, Colburn L (2000). Racial and ethnic disparities in faculty promotion in academic Medicine. JAMA.

[3] Ferrari L, Mari V, Parini S (2022). Discrimination toward women in surgery: a systematic scoping review. Ann Surg.

[4] Tseng ES, Williams BH, Santry HP (2022). History of equity, diversity, and inclusion in trauma surgery: for our patients, for our profession, and for ourselves. Curr Trauma Rep.

[5] Marinello F, Fleming CA, Möeslein G (2022). Diversity bias in colorectal surgery: a global perspective. Updates Surg.

[6] American College of Surgeons ACS, Diversity, Equity and Inclusion. https://www.facs.org/about-acs/diversity-equity-and-inclusion/. Accessed 17 Nov 2022

[7] American Society of Colon and Rectal Surgeons ASCRS, Diversity Equity and Inclusion Committee. https://fascrs.org/my-ascrs/leadership/committees?cid=DEI. Accessed 17 Nov 2022

[8] The Association of Coloproctology of Great Britain and Ireland ACPGBI, quality, Diversity and Inclusion (EDI) Committee. https://www.acpgbi.org.uk/about/committees/1005/equality_diversity_and_inclusion_edi_committee/public. Accessed 13 Dec 2022

[9] Keller DS, Berho M, Brown G (2020). A narrative celebrating the recent contributions of women to colorectal surgery. Surgery.

[10] Calise F, Spolverato G, Piccoli M (2021). Gender gap or gender bias? That is the question. Updates Surg.

[11] Heiderscheit EA, Schlick CJR, Ellis RJ (2022). Experiences of LGBTQ+ residents in US general surgery training programs. JAMA Surg.

[12] Au S, Bellato V, Carvas JM, Córdoba CD, Daudu D, Dziakova J, Eltarhoni K, El Feituri N, Fung ACH, Fysaraki C, Gallo G, Gultekin FA, Harbjerg JL, Hatem F, Ioannidis A, Jakobsen L, Clinch D, Kristensen HØ, Kuiper SZ, Kwok AMF, Kwok W, Millan M, Milto KM, Ng HJ, Pellino G, Picciariello A, Pronin S, van Ramshorst GH, Ramser M, Jiménez-Rodríguez RM, Sainz Hernandez JC, Samadov E, Sohrabi S, Uchiyama M, Wang JH, Younis MU, Fleming S, Alhomoud S, Mayol J, Moeslein G, Smart NJ, Soreide K, Teh C, Verran D, Maeda Y (2021). Global parental leave in surgical careers: differences according to gender, geographical regions and surgical career stages. Br J Surg.

[13] van Loon YT, Jiménez Rodríguez R, Keller DS (2021). Female representation and position based on facts and members views in the european society of coloproctology. Dis Colon Rectum.

[14] Lam K, Abràmoff BJM, Bishop SM, Brady RR, Callcut RA, Chand M, Collins JW, Diener MK, Eisenmann M, Fermont K, Neto MG, Hager GD, Hinchliffe RJ, Horgan A, Jannin P, Langerman A, Logishetty K, Mahadik A, Maier-Hein L, Antona EM, Mascagni P, Mathew RK, Müller-Stich BP, Neumuth T, Nickel F, Park A, Pellino G, Rudzicz F, Shah S, Slack M, Smith MJ, Soomro N, Speidel S, Stoyanov D, Tilney HS, Wagner M, Darzi A, Kinross JM, Purkayastha S (2022). A Delphi consensus statement for digital surgery. NPJ Digit Med..

[15] Farhan SA, Hayat J, Daniyal M, Ahmed SH, Karimuddin AA, Khosa F (2023). The virtual face of colon and rectal surgery training in the USA: an in-depth evaluation and analysis of fellowship programs website content. World J Surg.

[16] Tekkis NP, Richmond-Smith R, Pellino G, Kontovounisios C (2022). Facilitating the adoption and evolution of digital technologies through re-conceptualization. Front Surg.

[17] Viganò L, Giuliani A, Calise F (2019). The dilemma of surgical research between evidences and experience, impact factor and innovation. Updates Surg.

[18] NIHR Global Health Unit on Global Surgery; COVIDSurg Collaborative (2022). Elective surgery system strengthening: development, measurement, and validation of the surgical preparedness index across 1632 hospitals in 119 countries. Lancet.

[19] Crepaz L, Sartori A, Podda M, Ortenzi M, Di Leo A, Stabilini C, Carlucci M, Olmi S, SICE/ISHAWS collaborative group (2023). Minimally invasive approach to incisional hernia in elective and emergency surgery: a SICE (Italian Society of Endoscopic Surgery and new technologies) and ISHAWS (Italian Society of Hernia and Abdominal Wall Surgery) online survey. Updates Surg..

[20] Podda M, Pellino G, Di Saverio S, Coccolini F, Pacella D, Cioffi SPB, Virdis F, Balla A, Ielpo B, Pata F, Poillucci G, Ortenzi M, Damaskos D, De Simone B, Sartelli M, Leppaniemi A, Jayant K, Catena F, Giuliani A, Di Martino M, Pisanu A, MANCTRA-1 Collaborative Group (2023). Infected pancreatic necrosis: outcomes and clinical predictors of mortality. A post hoc analysis of the MANCTRA-1 international study. Updates Surg..

[21] ESCP Eagle Safe Anastomosis Collaborative (2021). ESCP Safe Anastomosis ProGramme in CoLorectal SurgEry (EAGLE): study protocol for an international cluster randomised trial of a quality improvement intervention to reduce anastomotic leak following right colectomy. Colorectal Dis.

[22] DAMASCUS Study Management Group (2021). Diverticulitis management, a snapshot collaborative audit study (DAMASCUS): protocol for an international, multicentre, prospective observational study. Colorectal Dis.

[23] Lagisz M, Aich U, Amin B, Rutkowska J, Sánchez-Mercado A, Lara CE, Nakagawa S (2023). Little transparency and equity in scientific awards for early- and mid-career researchers in ecology and evolution. Nat Ecol Evol.

[24] EuroSurg Collaborative (2016). EuroSurg: a new European student-driven research network in surgery. Colorectal Dis.

[25] EuroSurg Collaborative (2018). Body mass index and complications following major gastrointestinal surgery: a prospective, international cohort study and meta-analysis. Colorectal Dis. 20(8):215–225. 10.1111/codi.14292 10.1111/codi.14292 29897171

[26] El-Hussuna A, Rubio-Perez I, Millan M, Pellino G, Negoi I, Gallo G, Shalaby M, Celentano V, Green R, Minaya-Bravo A, Emile S, Smart NJ, Maeda Y, Ivatury SJ, Mackenzie G, Yalçınkaya A, Mellenthin C, Dudi-Venkata NN, Davies J, McNair A, Pata F, Gymoese Berthelsen K, Rivadeneira D, Spinelli A, Myrelid P, Mayol J, Wexner S, OpenSourceResearch collaboration (2021). Patient-reported outcome measures in colorectal surgery: construction of core measures using open-source research method. Surg Innov.

[27] Chowdhury S, El-Hussuna A, Gallo G, Keatley J, Kelly ME, Minaya-Bravo A, Ovington L, Pata F, Pellino G, Pinkney T, Sanchez Guillen L, Schmitz ND, Spychaj K, Riess C, van Ramshorst GH, 2021 European Society of Coloproctology (ESCP) collaborating group (2023). An international assessment of surgeon practices in abdominal wound closure and surgical site infection prevention by the European society for coloproctology. Colorectal Dis.

[28] Tripartite Gastrointestinal Recovery Post-operative Ileus Group (2022). Core outcome set for clinical studies of postoperative ileus after intestinal surgery. Br J Surg.

[29] Northington GM, Acevedo-Alvarez MG, Willis-Gray MG (2022). The American urogynecologic society action plan on diversity, equity, and inclusion: developed by the diversity, equity, and inclusion task force. Female Pelvic Med Reconstr Surg.

[30] Aulivola B, Mitchell EL, Rowe VL, Smeds MR (2021). Ensuring equity, diversity, and inclusion in the society for vascular surgery: a report of the society for vascular surgery task force on equity, diversity, and inclusion. J Vasc Surg.

[31] Seehra J, Lewis-Lloyd C, Koh A (2021). Publication rates, ethnic and sex disparities in UK and Ireland surgical research prize presentations: an analysis of data from the Moynihan and Patey prizes from 2000 to 2020. World J Surg.

[32] West-Livingston LN, Dittman JM, Park JA (2021). Sexual orientation, gender identity, and gender expression: From current state to solutions for the support of lesbian, gay, bisexual, transgender, and queer/questioning patients and colleagues. J Vasc Surg.

[33] Kim Y, Al-Faraaz K, McElroy IE (2022). The current status of the diversity pipeline in surgical training. Am J Surg.

[34] Gowda S (2021). Flattening the surgical field: ensuring diversity and maximizing inclusion within surgical specialties. BMJ.

[35] O’Flynn E, Danial A, Gajewski J (2021). Global surgery education and training programmes—a scoping review and taxonomy. Indian J Surg.

